# Checklist of the helminth parasites of the genus *Profundulus* Hubbs, 1924 (Cyprinodontiformes, Profundulidae), an endemic family of freshwater fishes in Middle-America

**DOI:** 10.3897/zookeys.523.6088

**Published:** 2015-09-28

**Authors:** Carlos Daniel Pinacho-Pinacho, Martín García-Varela, Jesús S. Hernández-Orts, Carlos A. Mendoza-Palmero, Ana L. Sereno-Uribe, Emilio Martínez-Ramírez, Leopoldo Andrade-Gómez, Alejandra López-Jiménez, Eduardo Hernández-Cruz, Gerardo Pérez-Ponce de León

**Affiliations:** 1 Departamento de Zoología, Instituto de Biología, Universidad Nacional Autónoma de México, Apartado Postal 70-153, C. P. 14510, México, D. F., México; 2 Posgrado en Ciencias Biológicas, Instituto de Biología, Universidad Nacional Autónoma de México, Apartado Postal 70-153, C.P. 04510, México, D.F., México; 3 Departamento de Investigación, Área de Acuacultura, Centro Interdisciplinario de Investigación para el Desarrollo Integral Regional, Unidad Oaxaca, Instituto Politécnico Nacional. Hornos Núm. 1003, Col. Noche Buena, Santa Cruz Xoxocotlán, 71230 Oaxaca, México

**Keywords:** Killifish, Profundulidae, Middle-America, Digenea, Monogenea, Cestoda, Nematoda

## Abstract

From December 2012 to November 2014, 267 fish belonging to the family Profundulidae (representing nine of the 11 species of the genus *Profundulus*) were collected in 26 localities of Middle-America, across southern Mexico, Guatemala, and Honduras, comprising the distribution range of the genus, and analyzed for helminth parasites. Additionally, a database with all ten available published accounts of the helminth parasite fauna of this genus (the only genus within the family) was assembled. Based on both sources of information, a checklist containing all the records was compiled as a tool to address future questions in the areas of evolutionary biology, biogeography, ecology and phylogeography of this host-parasite association. The helminth parasite fauna of this fish group consists of 20 nominal species, classified in 17 genera and 14 families. It includes six species of adult digeneans, five metacercariae, two monogeneans, one adult cestode, three adult nematodes and three larval nematodes. The profundulid fishes are parasitized by a specialized group of helminth species (*e.g.* *Paracreptotrema blancoi* *sensu* Salgado-Maldonado et al. (2011b), *Paracreptotrema profundulusi* Salgado-Maldonado, Caspeta-Mandujano & Martínez Ramírez, 2011, *Phyllodistomum spinopapillatum* Pérez-Ponce de León, Pinacho-Pinacho, Mendoza-Garfias & García-Varela, 2015, *Spinitectus humbertoi* Mandujano-Caspeta & Moravec, 2000, *Spinitectus mariaisabelae* Caspeta-Mandujano Cabañas-Carranza & Salgado-Maldonado, 2007 and *Rhabdochona salgadoi* Mandujano-Caspeta & Moravec, 2000), representing the core helminth fauna that are not shared with other Middle-American fish species.

## Introduction

The information gathered regarding the composition of the helminth parasites of freshwater fishes of Mexico has increased in recent years (Pérez-Ponce de León and Choudhury 2010). The large number of published papers contributing to the inventory of the helminth parasite fauna of fish hosts in the last decades allowed Luque and Poulin (2007) to suggest that Mexico stands out as a hotspot of parasite diversity in freshwater fishes. The species composition of the helminth fauna of some freshwater fish families, such as the Cichlidae and Goodeidae, is well known (Vidal-Martínez et al. 2001; Martínez-Aquino et al. 2014).

The distribution of the Profundulidae extends along the Atlantic and Pacific Ocean slopes of southern Mexico, Guatemala, El Salvador and Honduras (Miller 1955; Miller et al. 2005; Doadrio et al. 1999; Matamoros and Schaeffer 2010; Matamoros et al. 2012); from the Río Aguacatillo (a tributary of the Laguna Tres Palos) in Guerrero, Mexico to the Río Nacaome in Honduras, and on the Atlantic slope from the Río Quiotepec (the Río Papaloapan drainage basin) in Oaxaca, to the Río Ulúa, Honduras (Martínez-Ramírez et al. 2004; Matamoros et al. 2012). The family contains a single genus (*Profundulus* Hubbs, 1924), the current species composition of which is a matter of debate, since some authors recognize only eight valid species (Matamoros and Schaeffer 2010; Matamoros et al. 2012), whereas others (see Doadrio et al. 1999: Martínez-Ramírez et al. 2004) also recognize *Profundulus balsanus* Ahl, 1935 as a valid species, as well as two undescribed taxa, *Profundulus* sp. 1, and *Profundulus* sp. 2, which are currently being described by one of us (EMR). In addition to this, a molecular analysis of nuclear and mitochondrial genes, which will be published elsewhere, corroborates the validity of these three species (Ornelas-García, pers. comm.). Irrespective of the species composition, all *Profundulus* species represent an endemic lineage in Middle-America that has probably inhabited this region since the Pliocene and perhaps even the Miocene (Miller 1955; Doadrio et al. 1999; González-Diaz et al. 2005; Matamoros and Schaeffer 2010).

Records of the helminth parasite fauna of this family began with Caspeta-Mandujano and Moravec (2000), who described two nematode species, *Spinitectus humbertoi* Mandujano-Caspeta & Moravec, 2000 and *Rhabdochona salgadoi* Caspeta-Mandujano & Moravec, 2000, from the intestine of *Profundulus labialis* (Günther, 1866) in Inzcuinatoyac, Guerrero, Mexico. To date, ten studies have been published regarding some aspects of the helminth parasite fauna of profundulids, including descriptions of new species, inventories in particular localities and analyses of the parasite community structure of particular host species (Caspeta-Mandujano et al. 2007; Velázquez-Velázquez et al. 2011; Salgado-Maldonado et al. 2011a, b; Pinacho-Pinacho et al. 2014; Salgado-Maldonado et al. 2014, 2015; Velazquez-Velazquez et al. 2015; Pérez-Ponce de León et al. 2015).

As a continuation of effort to provide a more complete inventory of the helminth parasite fauna of freshwater fishes in this geographical region, intensive samplings were conducted during the last few years of these killifishes throughout their ranges of distribution in an attempt to obtain empirical and robust data to enable further studies of this host-parasite association from a phylogenetic, phylogeographical and biogeographical perspective, with the aim of understanding the mechanisms that have shaped the evolutionary and biogeographical history of these fishes and their parasites in Middle-America. The main objectives of this research were (1) to compile all the available published accounts on the helminth fauna of profundulid fishes, and (2) to incorporate new data derived from our own survey work of the last few years.

## Material and methods

**Data compilation**. A dataset of all published records of the helminth species reported from members of the family Profundulidae in Middle-America was compiled. The keywords “Parasit(e)*AND *Profundulus*” were used to conduct searches through the ISI Web of Science. All those studies whose datasets provided taxonomic information on the helminth taxa found in a sample of individual hosts were considered.

**Figure 1.**
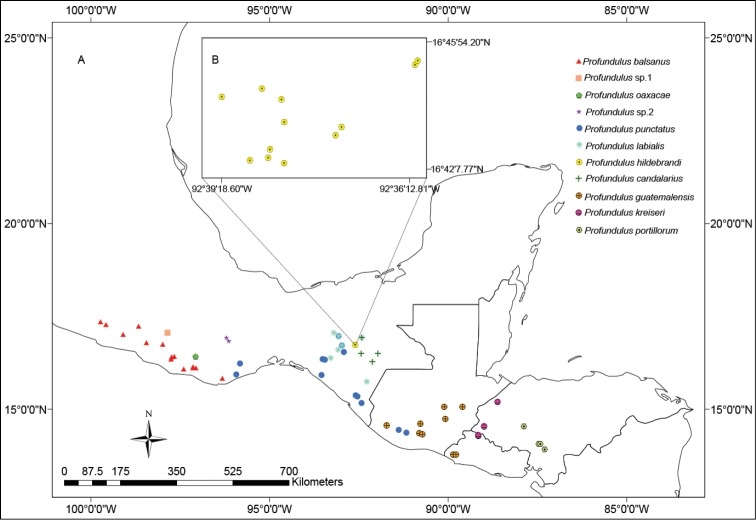
**A** Map of Middle-America indicating the localities where specimens of Profundulidae have been have been examined for helminth parasites. Colors and symbols correspond for each species of *Profundulus* **B** Zoom of an endorrheic basin in San Cristóbal de la Casas, Chiapas, Mexico showing localities where the endemic fish *Profundulus hildebrandi* was collected.

**Current research**. Original data from our own studies of the last two years were included. From December 2012 through to November 2014, 267 individual fish belonging to nine species of *Profundulus*, i.e. *Profundulus balsanus*; *Profundulus candalarius* Hubbs, 1924; *Profundulus guatemalensis* (Günther, 1866); *Profundulus hildebrandi* Miller, 1950; *Profundulus kreiseri* Matamoros, Schaefer, Hernández & Chakrabarty, 2012; *Profundulus labialis*; *Profundulus portillorum* Matamoros & Schaefer, 2010; *Profundulus punctatus* (Günther, 1866); and *Profundulus* sp. 2 (*sensu* Doadrio et al. 1999), were examined for helminth parasites. Fish were collected with seine nets and electrofishing in 26 localities of southern Mexico, Guatemala and Honduras (Fig. 1; Table 1). Fish were kept alive and examined for helminths no more than 4 h after their capture. Fish were killed with an overdose of anesthetic and placed in Petri dishes, and immediately examined for helminths. All the external surfaces, viscera and musculature of each host were examined under a stereomicroscope, and the helminths found were counted *in situ*. Adult digeneans and metacercariae, monogeneans, cestodes and nematodes were fixed in hot 4% neutral formalin, and additional specimens were fixed in 100% ethanol for future molecular studies. Some monogeneans were mounted in a mixture of glycerine-ammonium picrate (Ergens 1969) and in Gray-Wess medium (Vidal-Martínez et al. 2001) to study their sclerotized structures. Digeneans, cestodes and monogeneans used for morphological studies were stained with Mayer’s paracarmine or iron acetocarmine, dehydrated using a graded alcohol series, cleared in methyl salicylate and mounted as permanent slides in Canada balsam. Nematodes were cleared with glycerine for light microscopy and stored in 70% ethanol. Voucher specimens of some helminth species were deposited in the Colección Nacional de Helmintos (CNHE), Instituto de Biología, Universidad Nacional Autónoma de México, Mexico City, Mexico. Additionally, vouchers of *Bothriocephalus acheilognathi* Yamaguti, 1934 were deposited in the Helminthological Collection of the Institute of Parasitology (IPCAS), Biology Centre ASCR, České Budějovice, Czech Republic (accession numbers IPCAS C-15). Parameters of infection such as prevalence (% infected) and mean intensity of infection (the average number of a particular species of parasite among the infected members of a particular host species) were calculated following Bush et al. (1997).

**Table 1.** Localities in Mexico, Guatemala, El Salvador and Honduras where at least one helminth species has been recorded as a parasite of *Profundulus*. Localities marked with an asterisk (*) were sampled in this study. Collection sites (CS), locality (four letters code), geographical coordinates, country sampled and references are included. The collection site (CS) and locality code correspond with the localities referred in Table 2.

CS	Locality (code)	Geographical coordinates		Country	References
		N	W		
(1)	Arroyo Inzcuinatoyac (Inzc)	17°21'39"	99°44'00"	México	Caspeta-Mandujano and Moravec (2000)
(2)	Río Suchiapa (Such)	16°20'06"	93°27'19"	México	Caspeta-Mandujano et al. (2007)
(3)	Ecosur (Ecos)	16°42'55"	92°37'28"	México	Velázquez-Velázquez et al. (2011)
(4)	La Albarrada (Alba)	16°42'37"	92°37'32"	México	Velázquez-Velázquez et al. (2011)
(5)	5 de Marzo (5mar)	16°42'34"	92°38'14"	México	Velázquez-Velázquez et al. (2011)
(6)	El Puente (Puen)	16°43'59"	92°36'54"	México	Velázquez-Velázquez et al. (2011)
(7)	Arroyo Chamula (Cham)	16°44'52"	92°39'22"	México	Velázquez-Velázquez et al. (2011)
(8)	Peje de Oro (Peor)	16°44'48"	92°37'00"	México	Velázquez-Velázquez et al. (2011)
(9)	El Arcotete (Arco)	16°45'57"	92°31'43"	México	Velázquez-Velázquez et al. (2011)
(10)	Arenal (Aren)	16°43'31"	92°34'53"	México	Velázquez-Velázquez et al. (2011)
(11)	Agua de Pajarito (Paja)	16°43'43"	92°34'44"	México	Velázquez-Velázquez et al. (2011)
(12)	Laguna Soyul (Lsoy)	16°46'01"	92°31'39"	México	Velázquez-Velázquez et al. (2011)
(13)	Río Ocotlán (Ocot)	-	-	México	Salgado-Maldonado et al. (2011a)
(14)	Río Chicomosuelo (Chic)	15°44'38"	92°16'50"	México	Salgado-Maldonado et al. (2011a)
(15)	Río Suchiapa, José María Garza (Rsuc)	16°36'36"	93°05'03"	México	Salgado-Maldonado et al. (2011a)
(16)	Río San Juan, puente El Tablón, Villa Flores (Saju)	16°21'01"	93°30'56"	México	Salgado-Maldonado et al. (2011a)
(17)	Piedra Labrada (Labr)	18°58'54"	99°14'12"	México	Salgado-Maldonado et al. (2011b)
(18)	Río La Soledad Carrizo (Carr)	16°25'0.4"	97°40'12.9"	México	Salgado-Maldonado et al. (2011b) Pinacho-Pinacho et al. (2014)
(19)	Río San José de las Flores (Flor)	16°24'21.5"	97°44'22.6"	México	Salgado-Maldonado et al. (2011b) Pinacho-Pinacho et al. (2014)
(20)	Río Santa Cruz Flores Magón (Fmag)*	16°21'6.1"	97°45'38.3"	México	Salgado-Maldonado et al. (2011b) Pinacho-Pinacho et al. (2014) Pérez-Ponce de León et al. (2015) **This study**
(21)	Río Pichuaca (Pich)*	16°05'34.2"	97°24'18.1"	México	Salgado-Maldonado et al. (2011b) Pinacho-Pinacho et al. (2014) **This study**
(22)	Río La Reforma (Refo)	16°08'33.5"	97°08'41.6"	México	Salgado-Maldonado et al. (2011b) Pinacho-Pinacho et al. (2014)
(23)	Río Pueblo Viejo (Viej)*	16°06'22.3"	97°03'47.8"	México	Salgado-Maldonado et al. (2011b) Pinacho-Pinacho et al. (2014) Pérez-Ponce de León et al. (2015) **This study**
(24)	Río Santa María Huatulco (Huat)*	15°50'14.2"	96°19'30.8"	México	Salgado-Maldonado et al. (2011b) Pinacho-Pinacho et al. (2014) **This study**
(25)	Río Macuta (Macu)	-	-	México	Salgado-Maldonado et al. (2011b)
(26)	Río Templo, San Juan del Río (Sjri)*	16°53'56.3"	96°09'57.3"	México	Salgado-Maldonado et al. (2011b) Pérez-Ponce de León et al. (2015) **This study**
(27)	Arroyo Ojo de Agua (Ojag)	16°13'38.6"	95°49'36.6"	México	Salgado-Maldonado et al. (2011b)
(28)	Río La Laca (Rlac)	17°14'09.3"	98°39'55.7"	México	Salgado-Maldonado et al. (2014)
(29)	Río Cahoapan (Caho)	17°16'37.8"	99°35'04.7"	México	Salgado-Maldonado et al. (2014)
(30)	Río Tamarindo (Tama)	17°00'36.5"	99°06'0.8"	México	Salgado-Maldonado et al. (2014)
(31)	Río del Aguacate (Agua)	16°07'19"	97°8'22.8"	México	Salgado-Maldonado et al. (2014)
(32)	Arroyo los Sabinos (Sabi)	16°25'39.9"	97°4'28.9"	México	Salgado-Maldonado et al. (2014)
(33)	Río Chacalapa (Chac)	15°55'54.8"	95°56'00.3"	México	Salgado-Maldonado et al. (2014)
(34)	Río Chicaguaxtla (Chic)	17°03'30.30"	97°51'32.52"	México	Salgado-Maldonado et al. (2014)
(35)	Río Chico (Rchi)	16°55'34.50"	96°12'27.42"	México	Salgado-Maldonado et al. (2014)
(36)	Amatenango del Río (Amri)	16°31'22.2"	92°25'10.7"	México	Salgado-Maldonado et al. (2014)
(37)	Río Grande de Comitán (Rcom)	16°16'49.9"	92°07'21.1"	México	Salgado-Maldonado et al. (2014)
(38)	Arroyo ECOSUR (Aeco)	16°42'27.3"	92°36'54.8"	México	Salgado-Maldonado et al. (2014)
(39)	Arroyo Teopisca (Ateo)	16°33'13.7"	92°28'34.9"	México	Salgado-Maldonado et al. (2014) Velazquez-Velazquez et al. (2015)
(40)	Río Rancho San Antonio (Rsan)	16°58'30.9"	93°03'44.7"	México	Salgado-Maldonado et al. (2014)
(41)	Arroyo Tres Picos (Atpi)	17°03'28.3"	93°11'50.7"	México	Salgado-Maldonado et al. (2014)
(42)	Río Nandalumi (Rnan)	16°43'18.4"	92°58'52.4"	México	Salgado-Maldonado et al. (2014)
(43)	Arroyo Ojo de Agua, El Canelar (Cane)	16°32'08"	92°55'02.5"	México	Salgado-Maldonado et al. (2014)
(44)	Río Nil (Rnil)	14°33'54.4"	91°43'25.4"	Guatemala	Salgado-Maldonado et al. (2014)
(45)	Río el Cantil (Cant)	14°21'22.6"	90°48'30.4"	Guatemala	Salgado-Maldonado et al. (2014)
(46)	Arroyo El Platanar (Apla)	14°36'58.6"	90°46'37.9"	Guatemala	Salgado-Maldonado et al. (2014)
(47)	Río Cauca (Rcau)	13°46'42.6"	89°51'40.8"	El Salvador	Salgado-Maldonado et al. (2014)
(48)	Río Cauca (Cauc)	13°46'41.6"	89°46'41.67"	El Salvador	Salgado-Maldonado et al. (2014)
(49)	Quebrada Los Tecomates (Qtec)	14°18'11.3"	89°09'40.8"	El Salvador	Salgado-Maldonado et al. (2014)
(50)	Río Nonoalpa (Nono)	14°17'23.2"	89°09'10.7"	El Salvador	Salgado-Maldonado et al. (2014)
(51)	Río Ojojona (Ojoj)	13°55'43.7"	87°17'40"	Honduras	Salgado-Maldonado et al. (2014)
(52)	Lepaterique (Lepa)	14°03'42.9"	87°27'58.6"	Honduras	Salgado-Maldonado et al. (2014)
(53)	Lepaterique (1Lep)	14°04'14.4"	87°25'56.9"	Honduras	Salgado-Maldonado et al. (2014)
(54)	El Platanar, Putla de Guerrero (Plat)*	16°44´55˝	97°59´32˝	México	**This study**
(55)	Río San José, Santiago Jamiltepec (Jami)*	16°24´19˝	97°44´20˝	México	**This study**
(56)	Río San Juan, Cristobal Obregón (Obre)*	16°21´00˝	93°30´54˝	México	**This study**
(57)	Río Suchiapan, La Esperanza (Espe)*	16°23´27.60˝	93°17´24˝	México	**This study**
(58)	Río Pedregal, Tres Picos (Tpic)*	15°55´1.2˝	93°32´45.6˝	México	**This study**
(59)	Río Huixtla (Huix)*	15°10´18˝	92°25´24˝	México	**This study**
(60)	El Triunfo (Triu)*	15°20´44˝	92°32´30˝	México	**This study**
(61)	Río Nueva Francia (Fran)*	15°22´7.58˝	92°35´20.2˝	México	Pérez-Ponce de León et al. (2015) **This study**
(62)	Río Nahualate (Nahu)*	14°26´44˝	91°22´56˝	Guatemala	**This study**
(63)	Río Primavera (Prim)*	14°22´19.20˝	91°09´60˝	Guatemala	**This study**
(64)	Río Escuintla (Escu)*	14°19´41.51˝	91°42´57.35˝	Guatemala	**This study**
(65)	Río Las Cabezas, Saranate (Sara)*	14°44´23˝	90°04´52˝	Guatemala	**This study**
(66)	Puente Sansare (Sans)*	14°44´52˝	90°06´33˝	Guatemala	**This study**
(67)	Río Hondo (Rhon)*	15°03´55.50˝	89°35´48.28˝	Guatemala	**This study**
(68)	Arroyo en Hidroeléctrica Chamelecón (Cham)*	15°11´51.60˝	89°36´57.60˝	Honduras	**This study**
(69)	Quebrada El Paraiso (Qpar)*	15°01´26˝	88°59´32˝	Honduras	**This study**
(70)	Los Potrerillos (Lpot)*	14°32´31˝	87°52´55˝	Honduras	**This study**
(71)	Río San Carlos (Rcar)*	16°19´10˝	91°58´06˝	México	**This study**
(72)	Río La Gloria (Lglo)*	16°30´01˝	92°26´01˝	México	**This study**
(73)	Arroyo Moxviquil (Moxv)*	16°54´9.00˝	92°37´50˝	México	**This study**
(74)	Arroyo Peje de Oro (Poro)*	16°44´48˝	92°36´60˝	México	**This study**

## Results

The data analysis of both the bibliographic information and the survey work shows that 11 species of *Profundulus* (including undescribed species) studied for helminths, and that the list of helminth parasites of fish of this genus consists of 20 species classified in two taxonomic groups: Platyhelminthes (six adult digeneans, five metacercariae, two monogeneans and one adult cestode) and Nematoda (three adults and three larvae). Interestingly, no acanthocephalans and no larval cestodes are part of the helminth fauna of this fish group across its geographical distribution. Most taxa were identified to species level, except for larval stages which lacked the diagnostic characteristics present only in adult forms which are found in fish-eating birds (or freshwater turtles in the case of *Spiroxys* sp.). To better visualize the information from the checklist, the results are presented in two tables. Table 2 shows a parasite-host list. Species of parasites are organized by developmental stage, either as adults or larvae, and ordered alphabetically by family name. Species within each family are then listed alphabetically followed by their authority. The host-parasite list (Table 3) is organized alphabetically. Within each fish species, helminth parasites are listed alphabetically by taxonomic group, with their developmental stage indicated in parentheses.

**Table 2.** Parasite-host list of *Profundulus* in Middle-America. Locality abbreviations (CS-Code) correspond to those in Table 1. Key: N = number of examined hosts in each study, Site(s) of infection, P = Prevalence, MI ± SD = Mean Intensity ± standard deviation, CNHE = catalog numbers of specimens deposited in the collection.

Helminth taxa	Host (s)	Locality (CS-Code)	N	Site (s) of infection	P (%)	MI±SD	CNHE (Number of specimens)	Reference
**Adult Trematoda**								
**Family Allocreadiidae Looss, 1902**								
***Paracreptotrema blancoisensu*Salgado-Maldonado et al. (2011b).** (Fig. 2A).	*Profundulus balsanus*	(17-Labr)	29	Intestine	50	4.8 ± 4.2	7687 (15)	Salgado-Maldonado et al. (2011b)
		(18-Carr)	25	Intestine	8	1.0 ± 0	7688 (1)	Salgado-Maldonado et al. (2011b) Pinacho-Pinacho et al. (2014)
		(19-Flor)	20	Intestine	25	1.0 ± 0	7689 (3)	Salgado-Maldonado et al. (2011b) Pinacho-Pinacho et al. (2014)
		(20-Fmag)	18	Intestine	44.4	2.2 ± 1.03	7690 (4)	Salgado-Maldonado et al. (2011b) Pinacho-Pinacho et al. (2014)
			8	Intestine	75	1.5 ± 0.5		**This study**
		(21-Pich)	22	Intestine	59	2.0 ± 1.3	7691 (12)	Salgado-Maldonado et al. (2011b) Pinacho-Pinacho et al. (2014)
			4	Intestine	100	1.5 ± 0.5		**This study**
		(22-Refo)	20	Intestine	20	1.7 ± 0.9	7692 (3)	Salgado-Maldonado et al. (2011b) Pinacho-Pinacho et al. (2014)
		(23-Viej)	20	Intestine	10.0	1.5 ± 0.7	7686 (1)	Salgado-Maldonado et al. (2011b) Pinacho-Pinacho et al. (2014)
			10	Intestine	70	6.4 ± 8.8		**This study**
		(24-Huat)	7	Intestine	71.4	1.8 ± 0.4	7694 (3)	Salgado-Maldonado et al. (2011b) Pinacho-Pinacho et al. (2014)
			5	Intestine	-	-		**This study**
		(29-Caho)		Intestine	NR	NR		Salgado-Maldonado et al. (2014)
		(30-Tama)		Intestine	NR	NR		Salgado-Maldonado et al. (2014)
		(31-Agua)		Intestine	NR	NR		Salgado-Maldonado et al. (2014)
		(54-Plat)	4	Intestine	100	4 ± 4.2		**This study**
		(55-Jami)	7	Intestine	57.14	1.2 ± 0.5		**This study**
	*Profundulus oaxacae*	(25-Macu)	37	Intestine	37.8	2.6 ± 2.1	7693 (5)	Salgado-Maldonado et al. (2011b)
		(32-Sabi)		Intestine	NR	NR	9286 (2)	Salgado-Maldonado et al. (2014)
	*Profundulus* sp. 2	(26-Sjri)	43	Intestine	30.2	2.5 ± 2.2	7684 (7)	Salgado-Maldonado et al. (2011b)
			2	Intestine	100	3.5 ± 0.7		**This study**
		(35-Rchi)		Intestine	NR	NR		Salgado-Maldonado et al. (2014)
	*Profundulus punctatus*	(27-Ojag)	30	Intestine	6.6	5.5 ± 4.9	7685 (4)	Salgado-Maldonado et al. (2011b)
		(33-Chac)		Intestine	NR	NR		Salgado-Maldonado et al. (2014)
		(42-Rnan)		Intestine	NR	NR		Salgado-Maldonado et al. (2014)
		(56-Obre)	8	Intestine	50	3 ± 1.4		**This study**
		(58-Tpic)	15	Intestine	13.33	3.5 ± 0.7		**This study**
		(59-Huix)	20	Intestine	20	2.25 ± 1.8	9803 (2)	**This study**
		(60-Triu)	6	Intestine	33.33	1.5 ± 0.7	9804 (2)	**This study**
		(61-Fran)	15	Intestine	6.66	NR		**This study**
		(62-Nahu)	1	Intestine	100	1 ± 0		**This study**
		(63-Prim)	9	Intestine	66.66	6.25 ± 3.4		**This study**
	*Profundulus guatemalensis*	(44-Rnil)		Intestine	NR	NR		Salgado-Maldonado et al. (2014)
		(45-Cant)		Intestine	NR	NR		Salgado-Maldonado et al. (2014)
		(47-Rcau)		Intestine	NR	NR		Salgado-Maldonado et al. (2014)
		(64-Escu)	19	Intestine	50	2.1 ± 1.5		**This study**
		(65-Sara)	1	Intestine	100	1 ± 0		**This study**
		(66-Sans)	6	Intestine	100	4.8 ± 4.6		**This study**
	*Profundulus kreiseri*	(49-Qtec)		Intestine	NR	NR		Salgado-Maldonado et al. (2014)
		(69-Qpar)	28	Intestine	14.28	1.75 ± 1.5		**This study**
	*Profundulus labialis*	(57-Espe)	15	Intestine	6.66	NR		**This study**
	*Profundulus portillorum*	(70-Lpot)	9	Intestine	11.11	1 ± 0		**This study**
	*Profundulus candalarius*	(71-Rcar)	14	Intestine	78.57	4.3 ± 4.9		**This study**
Remarks: Specimens of *Paracreptotrema blancoi* *sensu* Salgado-Maldonado et al. (2011b) represent an undescribed species, but they require further taxonomic work.								
***Paracreptotrema profundulusi* Salgado-Maldonado, Caspeta-Mandujano & Martínez Ramírez, 2011.** (Fig. 2B).	*Profundulus* sp. 2	(26-Sjri)	43	Intestine	55.8	4 ± 5.7	7680 (1) 7681 (23)	Salgado-Maldonado et al. (2011b)
			2	Intestine	100	3.5 ± 0.7	9805 (1)	**This study**
		(35-Rchi)	NR	Intestine	NR	NR	9287 (1)	Salgado-Maldonado et al. (2014)
	*Profundulus punctatus*	(27-Ojag)	30	Intestine	20	6.8 ± 13.8	7682 (6)	Salgado-Maldonado et al. (2011b)
		(33-Chac)	NR	Intestine	NR	NR		Salgado-Maldonado et al. (2014)
	*Profundulus balsanus*	(19-Flor)	20	Intestine	5	4 ±0	7683 (4)	Salgado-Maldonado et al. (2011b) Pinacho-Pinacho et al. (2014)
		(29-Caho)	NR	Intestine	NR	NR		Salgado-Maldonado et al. (2014)
	*Profundulus oaxacae*	(32-Sabi)	NR	Intestine	NR	NR	9288 (1)	Salgado-Maldonado et al. (2014)
**Allocreadiidae gen. sp.**	*Profundulus portillorum*	(70-Lpot)	9	Intestine	11.11	1 ± 0		**This study**
Remarks: A single specimen was collected for future molecular studies.								
**Family Gorgoderidae Looss, 1901**								
***Phyllodistomum inecoli* Razo-Mendivil, Pérez-Ponce de León & Rubio-Godoy, 2013.** (Fig. 2C).	*Profundulus* sp. 2	(26-Sjri)	2	Urinary bladder	50	NR		Pérez-Ponce de León et al. (2015)
	*Profundulus punctatus*	(56-Obre)	15	Urinary bladder	NR	NR		Pérez-Ponce de León et al. (2015)
		(61-Fran)	15	Urinary bladder	6.66	NR	9302 (1)	Pérez-Ponce de León et al. (2015)
	*Profundulus candalarius*	(71-Rcar)	14	Urinary bladder	7.14	1 ± 0	9802 (1)	**This study**
		(72-Lglo)	22	Urinary bladder	31.81	1.5 ± 0.7	9661 (1)	**This study**
Remarks: This species was originally recorded by Pérez-Ponce de León et al. (2015) in fishes of genus *Profundulus*.								
***Phyllodistomum spinopapillatum* Pérez-Ponce de León, Pinacho-Pinacho, Mendoza-Garfias & García-Varela, 2015.** (Fig. 2D).	*Profundulus balsanus*	(18-Carr)	25	Urinary bladder	20	1 ±0	9667 (5)	Pinacho-Pinacho et al. (2014)
		(20-Fmag)	18	Urinary bladder	5.55	1 ±0	9666 (1)	Pinacho-Pinacho et al. (2014) Pérez-Ponce de León et al. (2015)
			8	Urinary bladder	25	1 ± 0	9671 (1)	**This study**
		(21-Pich)	22	Urinary bladder	4.54	1 ±0	9668 (1)	Pinacho-Pinacho et al. (2014)
		(22-Refo)	20	Urinary bladder	10	1 ±0		Pinacho-Pinacho et al. (2014)
		(23-Viej)	20	Urinary bladder	40	1.12 ±0.35	9299 (1) 9300 (7)	Pinacho-Pinacho et al. (2014) Pérez-Ponce de León et al. (2015)
			10	Urinary bladder	70	1.8 ± 01.5		**This study**
Remarks: This species was recorded as *Phyllodistomum* sp. by Pinacho-Pinacho et al. (2014). Posteriorly, based on morphological and molecular evidence this species was described as a new taxon by Pérez-Ponce de León et al. (2015).								
**Family Haploporidae Nicoll, 1914**								
***Saccocoelioides lamothei* Aguirre-Macedo & Violante-González, 2008.** (Fig. 2E).	*Profundulus balsanus*	(18-Carr)	25	Intestine	80	3.15 ±2.15	9806 (1)	Pinacho-Pinacho et al. (2014)
		(19-Flor)	20	Intestine	30	1.5 ±0.83		Pinacho-Pinacho et al. (2014)
		(20-Fmag)	18	Intestine	11.11	1 ±0		Pinacho-Pinacho et al. (2014)
			8	Intestine	12.5	1 ± 0		**This study**
		(55-Jami)	7	Intestine	42.85	5.3 ± 2.5		**This study**
Remarks: Pinacho-Pinacho et al. (2014) recorded originally this species as Culuwiya cf. cichlidorum, but detailed the morphological evaluation of voucher specimens deposited in the CNHE and molecular data indicate that this specimens corresponding with *Saccocoelioides lamothei* (Andrade-Gómez 2015).								
**Larval Trematoda**								
**Family Clinostomidae Lühe, 1901**								
***Clinostomum* sp.** (Fig. 2F).	*Profundulus punctatus*	(13-Ocot)	12	Mesentery	8.3	1±0.0	7442 (1)	Salgado-Maldonado et al. (2011a)
		(60-Triu)	6	Mesentery	16.66	NR		**This study**
	*Profundulus balsanus*	(18-Carr)	25	Mesentery, Gills, eyes, Body cavity	40	2.7 ±1.94	9202 (5)	Pinacho-Pinacho et al. (2014)
		(20-Fmag)	18	Mesentery, Gills, eyes, Body cavity	5.55	1 ±0		Pinacho-Pinacho et al. (2014)
		(22-Refo)	20	Mesentery, Gills, eyes, Body cavity	10	1 ±0		Pinacho-Pinacho et al. (2014)
		(23-Viej)	20	Mesentery, Gills, eyes, Body cavity	10	1.5 ±0.70		Pinacho-Pinacho et al. (2014)
		(24-Huat)	7	Mesentery, Gills, eyes, Body cavity	42.85	1 ±0		Pinacho-Pinacho et al. (2014)
			5	Mesentery	NR	NR		**This study**
		(54-Plat)	4	Mesentery	25	3 ± 0		**This study**
		(55-Jami)	7	Mesentery	14.28	1 ± 0	9660 (1)	**This study**
	*Profundulus candalarius*	(71-Rcar)	14	Mesentery	21.42	2 ± 0		**This study**
Remarks: This species was recorded as *Clinostomum complanatum* by Salgado-Maldonado et al. (2011a). However, based on recent findings by Sereno-Uribe et al. (2013), the species *Clinostomum complanatum* is most likely not found in Mexico, and instead they would correspond with *Clinostomum* sp. but this needs to be determined by further molecular work.								
**Family Diplostomidae Poirier, 1886**								
**Diplostomidae gen. sp.** (Fig. 2G).	*Profundulus balsanus*	(18-Carr)	25	Mesentery	4	1 ±0		Pinacho-Pinacho et al. (2014)
		(22-Refo)	20	Mesentery	5	4 ±0		Pinacho-Pinacho et al. (2014)
***Posthodiplostomum minimum* MacCallum, 1921.** (Fig. 2H).	*Profundulus balsanus*	(22-Refo)	20	Mesentery	25	2.6 ±1.34		Pinacho-Pinacho et al. (2014)
		(23-Viej)	20	Mesentery	5	1 ± 0		Pinacho-Pinacho et al. (2014)
			10	Mesentery	10	2 ± 0	9807 (1)	**This study**
		(24-Huat)	7	Mesentery	14.28	4 ± 0		Pinacho-Pinacho et al. (2014)
			5	Mesentery	NR	NR		**This study**
**Family Heterophyidae Leiper, 1909**								
**Ascocotyle (Ascocotyle) felippei Travassos, 1928.** (Fig. 2I).	*Profundulus balsanus*	(18-Carr)	25	Heart	28	165.42 ±72.39	9199 (10)	Pinacho-Pinacho et al. (2014)
		(19-Flor)	20	Heart	20	23.75 ±21.96		Pinacho-Pinacho et al. (2014)
		(20-Fmag)	18	Heart	83.33	16.73 ±15.07		Pinacho-Pinacho et al. (2014)
		(21-Pich)	22	Heart	86.36	58.94 ±43.31	9200 (6)	Pinacho-Pinacho et al. (2014)
		(22-Refo)	20	Heart	60	7.25 ±10.48		Pinacho-Pinacho et al. (2014)
		(23-Viej)	20	Heart	95	61.84 ±77.81		Pinacho-Pinacho et al. (2014)
		(24-Huat)	7	Heart	14.28	6 ±0		Pinacho-Pinacho et al. (2014)
	*Profundulus punctatus*	(63-Prim)	9	Heart	11.11	NR		**This study**
***Centrocestus formosanus* Nishigori, 1924.** (Fig. 2J).	*Profundulus balsanus*	(20-Fmag)	18	Gills	72.22	12.15 ±21.57		Pinacho-Pinacho et al. (2014)
		(21-Pich)	22	Gills	100	821.63 ±417.59	9201 (3)	Pinacho-Pinacho et al. (2014)
		(22-Refo)	20	Gills	100	42.45 ±33.39		Pinacho-Pinacho et al. (2014)
		(23-Viej)	20	Gills	5.88	31 ±0		Pinacho-Pinacho et al. (2014)
		(24-Huat)	7	Gills	50	1.66 ±1.15		Pinacho-Pinacho et al. (2014)
	*Profundulus punctatus*	(62-Nahu)	1	Gills	100	3 ± 0		**This study**
		(63-Prim)	9	Gills	11.11	NR		**This study**
**Monogenea**								
**Family Gyrodactylidae van Beneden & Hesse, 1863**								
***Gyrodactylus* sp.** (Fig. 3A–D).	*Profundulus balsanus*	(19-Flor)	20	Fins	5	1 ±0		Pinacho-Pinacho et al. (2014)
		(22-Refo)	20	Fins	5	2 ±0		Pinacho-Pinacho et al. (2014)
		(23-Viej)	20	Fins	10	1 ±0		Pinacho-Pinacho et al. (2014)
Remarks: The limited number of specimens found precluded the specific identification of this species; however, based on their morphology they clearly represent members of *Gyrodactylus*.								
**Family Dactylogyridae Bychowsky, 1937**								
***Urocleidoides* sp.** (Fig. 3E, F).	*Profundulus balsanus*	(19-Flor)	20	Gills	5	1 ±0		Pinacho-Pinacho et al. (2014)
		(20-Fmag)	18	Gills	72.22	7.15 ±6.37		Pinacho-Pinacho et al. (2014)
		(21-Pich)	22	Gills	13.63	3 ±1.73		Pinacho-Pinacho et al. (2014)
		(22-Refo)	20	Gills	75	7 ±5.45		Pinacho-Pinacho et al. (2014)
		(23-Viej)	20	Gills	82.35	5.85 ±5.27		Pinacho-Pinacho et al. (2014)
		(24-Huat)	7	Gills	83.33	7.4 ±4.44		Pinacho-Pinacho et al. (2014)
	*Profundulus punctatus*	(62-Nahu)	1	Gills	100	1 ±		**This study**
	*Profundulus guatemalensis*	(64-Escu)	19	Gills	5.26	4 ±		**This study**
		(65-Sara)	1	Gills	100	1 ±		**This study**
Remarks: Pinacho-Pinacho et al. (2014) recorded this specie as *Salsuginus* sp.; however, a detailed morphological evaluation of these specimens confirmed that they belong to *Urocleidoides* Mizelle & Price, 1964 (*sensu* Kritsky et al. 1986).								
**Adult Cestoda**								
**Family Bothriocephalidae Blanchard, 1849**								
***Bothriocephalus acheilognathi* Yamaguti, 1934.** (Fig. 3G, H).	*Profundulus hildebrandi*	(3-Ecos)	234	Intestine	54	4.62±2.38	7617 (2)	Velázquez-Velázquez et al. (2011)
		(4-Alba)	168	Intestine	61	13.10±8.57		Velázquez-Velázquez et al. (2011)
		(5-5mar)	173	Intestine	59	4.35 ± 2.51		Velázquez-Velázquez et al. (2011)
		(6-Puen)	85	Intestine	2	1±0.00		Velázquez-Velázquez et al. (2011)
		(7-Cham)	126	Intestine	41	1.88±0.55		Velázquez-Velázquez et al. (2011)
		(8-Peor)	128	Intestine	71	6.34±2.48		Velázquez-Velázquez et al. (2011)
		(9-Arco)	64	Intestine	11	2.57±4.48		Velázquez-Velázquez et al. (2011)
		(10-Aren)	64	Intestine	6	1±0.00		Velázquez-Velázquez et al. (2011)
		(11-Paja)	141	Intestine	5	1.14±0.76		Velázquez-Velázquez et al. (2011)
		(12-Lsoy)	4	Intestine	100	4.50±4.04		Velázquez-Velázquez et al. (2011)
		(73-Moxv)	20	Intestine	20	11 ± 9.9		**This study**
		(74-Poro)	7	Intestine	42.85	NR		**This study**
	*Profundulus portillorum*	(51-Ojoj)	30	Intestine	NR	NR	9368	Salgado-Maldonado et al. (2015)
	*Profundulus guatemalensis*	(66-Sans)	6	Intestine	16.66	4 ±	9670 (1)	**This study**
	*Profundulus candalarius*	(72-Lglo)	22	Intestine	22.72	1.6 ± 1.1	9669 (1)	**This study**
		(39- Ateo)	NR	Intestine	NR	NR		Velazquez-Velazquez et al. (2015)
Remarks: Velázquez-Velázquez et al. (2011) first recorded species of tapeworm in *Profundulus hildebrandi*. In the present study, the Asian fish tapeworm *Bothriocephalus acheilognathi* was recorded for the first time in *Profundulus guatemalensis* and *Profundulus candalarius*.								
**Adult Nematoda**								
**Family Cystidicolidae Skrjabin, 1946**								
***Spinitectus humbertoi* Caspeta-Mandujano & Moravec, 2000.** (Fig. 4A, B).	*Profundulus balsanus*	(1-Inzc)	1	Intestine	100	NR	4028 (1) 4030 (2)	Caspeta-Mandujano and Moravec (2000)
		(23-Viej)	20	Intestine	10	6.5 ±3.53		Pinacho-Pinacho et al. (2014)
		(24-Huat)	7	Intestine	14.28	17 ±0		Pinacho-Pinacho et al. (2014)
			5	Intestine	NR	NR	9443 (5)	**This study**
		(28-Rlac)	NR	Intestine	NR	NR		Salgado-Maldonado et al. (2014)
		(29-Caho)	NR	Intestine	NR	NR		Salgado-Maldonado et al. (2014)
		(30-Tama)	NR	Intestine	NR	NR		Salgado-Maldonado et al. (2014)
	*Profundulus punctatus*	(33-Chac)	NR	Intestine	NR	NR		Salgado-Maldonado et al. (2014)
		(40-Rsan)	NR	Intestine	NR	NR		Salgado-Maldonado et al. (2014)
	*Profundulus* sp. 1	(34-Chic)	NR	Intestine	NR	NR		Salgado-Maldonado et al. (2014)
	*Profundulus labialis*	(40-Rsan)	NR	Intestine	NR	NR		Salgado-Maldonado et al. (2014)
		(41-Atpi)	NR	Intestine	NR	NR		Salgado-Maldonado et al. (2014)
		(42-Rnan)	NR	Intestine	NR	NR		Salgado-Maldonado et al. (2014)
	*Profundulus guatemalensis*	(44-Rnil)	NR	Intestine	NR	NR		Salgado-Maldonado et al. (2014)
		(45-Cant)	NR	Intestine	NR	NR		Salgado-Maldonado et al. (2014)
		(47-Rcau)	NR	Intestine	NR	NR		Salgado-Maldonado et al. (2014)
		(48-Cauc)	NR	Intestine	NR	NR		Salgado-Maldonado et al. (2014)
		(66-Sans)	6	Intestine	50	3.3 ± 2	9639 (5)	**This study**
	*Profundulus kreiseri*	(50-Nono)	NR	Intestine	NR	NR		Salgado-Maldonado et al. (2014)
	*Profundulus portillorum*	(70-Lpot)	9	Intestine	22.22	NR	9638 (5)	**This study**
	*Profundulus candalarius*	(71-Rcar)	14	Intestine	57.14	2.6 ± 2.5		**This study**
Remarks: This nematode was originally described from the intestine of *Profundulus labialis* in Guerrero, Mexico. Apparently, the type host was erroneously identified by Caspeta-Mandujano and Moravec (2000) because *Profundulus labialis* is not distributed in Guerrero state, and most like these authors examined *Profundulus balsanus*. In this study this species was recorded in two new host species.								
***Spinitectus mariaisabelae* Caspeta-Mandujano Cabañas-Carranza & Salgado-Maldonado, 2007**	*Profundulus punctatus*	(2-Such)	NR	Intestine	NR	NR	5781(1)5783 (6)	Caspeta-Mandujano et al. (2007)
		(16-Saju)	3	Intestine	100	3.3±2.0		Salgado-Maldonado et al. (2011a)
		(13-Ocot)	12	Intestine	100	5.7±2.9		Salgado-Maldonado et al. (2011a)
	*Profundulus labialis*	(14-Chic)	3	Intestine	100	4.0±3.0		Salgado-Maldonado et al. (2011a)
		(15-Rsuc)	24	Intestine	79.2	4.3±3.2		Salgado-Maldonado et al. (2011a)
		(13-Ocot)	3	Intestine	100	2.6±2.8		Salgado-Maldonado et al. (2011a)
**Family Rhabdochonidae Travassos, Artigas & Pereira, 1928**								
***Rhabdochona salgadoi* Caspeta-Mandujano & Moravec, 2000.** (Fig. 4C, D).	*Profundulus balsanus*	(1-Inzc)	1	Intestine	100	NR	4031 (1) 4033 (32)	Caspeta-Mandujano and Moravec (2000)
		(18-Carr)	25	Intestine	60	4.4 ±4.15		Pinacho-Pinacho et al. (2014)
		(19-Flor)	20	Intestine	70	5.71 ±4.95		Pinacho-Pinacho et al. (2014)
		(20-Fmag)	18	Intestine	83.33	4.46 ±3.11		Pinacho-Pinacho et al. (2014)
		(21-Pich)	22	Intestine	54.54	2.75 ±1.86		Pinacho-Pinacho et al. (2014)
		(22-Refo)	20	Intestine	95	8.05 ±3.99		Pinacho-Pinacho et al. (2014)
		(23-Viej)	20	Intestine	90	7.66 ±4.95		Pinacho-Pinacho et al. (2014)
		(24-Huat)	7	Intestine	100	18.57 ±10.84		Pinacho-Pinacho et al. (2014)
		(28-Rlac)	NR	Intestine	NR	NR		Salgado-Maldonado et al. (2014)
		(29-Caho)	NR	Intestine	NR	NR		Salgado-Maldonado et al. (2014)
		(30-Tama)	NR	Intestine	NR	NR		Salgado-Maldonado et al. (2014)
		(31-Agua)	NR	Intestine	NR	NR		Salgado-Maldonado et al. (2014)
	*Profundulus oaxacae*	(32-Sabi)	NR	Intestine	NR	NR		Salgado-Maldonado et al. (2014)
	*Profundulus punctatus*	(33-Chac)	NR	Intestine	NR	NR		Salgado-Maldonado et al. (2014)
		(40-Rsan)	NR	Intestine	NR	NR		Salgado-Maldonado et al. (2014)
		(13-Ocot)	12	Intestine	66.7	3.2±1.9		Salgado-Maldonado et al. (2011a)
		(43-Cane)	NR	Intestine	NR	NR		Salgado-Maldonado et al. (2014)
		(59-Huix)	20	Intestine	30	NR	9637 (5)	**This study**
		(62-Nahu)	1	Intestine	100	NR		**This study**
		(63-Prim)	9	Intestine	22.22	NR		**This study**
	*Profundulus labialis*	(15-Rsuc)	24	Intestine	58.3	2.6±1.7		Salgado-Maldonado et al. (2011a)
		(40-Rsan)	NR	Intestine	NR	NR		Salgado-Maldonado et al. (2014)
		(41-Atpi)	NR	Intestine	NR	NR		Salgado-Maldonado et al. (2014)
	*Profundulus* sp. 2	(35-Rchi)	NR	Intestine	NR	NR		Salgado-Maldonado et al. (2014)
		(13-Ocot)	3	Intestine	33.3	6.0±0		Salgado-Maldonado et al. (2011a)
	*Profundulus* sp. 1	(34-Chic)	NR	Intestine	NR	NR		Salgado-Maldonado et al. (2014)
	*Profundulus candalarius*	(37-Rcom)	NR	Intestine	NR	NR		Salgado-Maldonado et al. (2014)
		(71-Rcar)	14	Intestine	7.14	1 ±	9640 (5)	**This study**
		(72-Lglo)	22	Intestine	4.54	1 ±		**This study**
	*Profundulus guatemalensis*	(44-Rnil)	NR	Intestine	NR	NR		Salgado-Maldonado et al. (2014)
		(64-Escu)	19	Intestine	21.05	NR	9642 (5)	**This study**
		(65-Sara)	1	Intestine	100	NR		**This study**
		(67-Rhon)	6	Intestine	16.66	NR		**This study**
	*Profundulus kreiseri*	(49-Qtec)	NR	Intestine	NR	NR	9290 (5)	Salgado-Maldonado et al. (2014)
		(68-Cham)	6	Intestine	83.33	NR	9641 (5)	**This study**
		(69-Qpar)	28	Intestine	71.42	NR		**This study**
		(50-Nono)	NR	Intestine	NR	NR		Salgado-Maldonado et al. (2014)
	*Profundulus portillorum*	(52-Lepa)	NR	Intestine	NR	NR		Salgado-Maldonado et al. (2014)
**Larval Nematodes**								
**Family Anisakidae Railliet & Henry, 1912**								
***Contracaecum* sp.**	*Profundulus punctatus*	(13-Ocot)	12	Intestine	8.3	1.0±0		Salgado-Maldonado et al. (2011a)
		(59-Huix)	20	Mesentery	5	NR		**This study**
		(60-Triu)	6	Mesentery	16.66	NR	9808 (1)	**This study**
**Family Dioctophymatidae Railliet, 1915**								
***Eustrongylides* sp.** (Fig. 4E, F).	*Profundulus punctatus*	(13-Ocot)	12	Intestine	41.6	1.6±0.5		Salgado-Maldonado et al. (2011a)
	*Profundulus balsanus*	(19-Flor)	20	Mesentery	5	1 ±0		Pinacho-Pinacho et al. (2014)
		(20-Fmag)	18	Mesentery	16.66	2 ±1		Pinacho-Pinacho et al. (2014)
	*Profundulus candalarius*	(71-Rcar)	14	Mesentery	7.14	NR	9809 (1)	**This study**
**Family Gnathostomatidae Railliet, 1895**								
***Spiroxys* sp.**	*Profundulus portillorum*	(70- Lpot)	9	Intestine	11.11	NR	9810 (1)	**This study**

**Figure 2.**
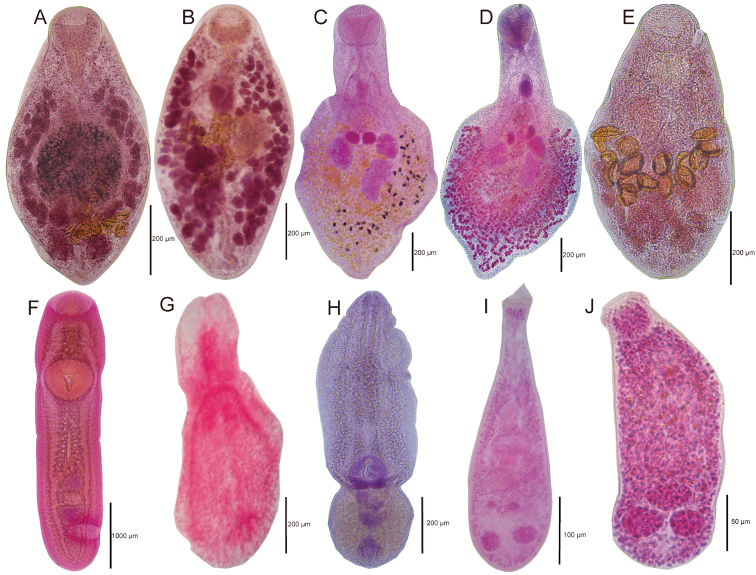
Species of trematodes found in *Profundulus* spp. **A** *Paracreptotrema blancoi* sensu Salgado-Maldonado et al. (2011b) **B** *Paracreptotrema profundulusi* **C** *Phyllodistomum inecoli* **D** *Phyllodistomum spinopapillatum* **E** *Saccocoelioides lamothei* **F** *Clinostomum* sp. **G** Diplostomidae gen. sp. **H** *Posthodiplostomum minimum* **I** Ascocotyle (Ascocotyle) felippei **J** *Centrocestus formosanus*.

**Table 3.** Host-parasite list. Key: A = Adult, M = Metacercariae, L = Larvae.

Host	Helminth parasite	Reference
*Profundulus balsanus* Ahl, 1935	**Digenea** Ascocotyle (Ascocotyle) felippei (M) *Centrocestus formosanus* (M) *Clinostomum* sp. (M) Diplostomidae gen. sp. (M) *Phyllodistomum spinopapillatum* (A) *Posthodiplostomum minimum* (M) *Paracreptotrema blancoi* (A) *Paracreptotrema profundulusi* (A) *Saccocoelioides lamothei* (A) **Monogenea** *Gyrodactylus* sp. (A) *Urocleidoides* sp. (A) **Nematoda** *Eustrongylides* sp. (L) *Rhabdochona salgadoi* (A) *Spinitectus humbertoi* (A)	Pinacho-Pinacho et al. (2014) Pinacho-Pinacho et al. (2014) Pinacho-Pinacho et al. (2014) **This study** Pinacho-Pinacho et al. (2014) Pinacho-Pinacho et al. (2014) Pérez-Ponce de León et al. (2015) **This study** Pinacho-Pinacho et al. (2014) **This study** Salgado-Maldonado et al. (2011b) Pinacho-Pinacho et al. (2014) Salgado-Maldonado et al. (2014) **This study** Salgado-Maldonado et al. (2011b) Pinacho-Pinacho et al. (2014) Salgado-Maldonado et al. (2014) Pinacho-Pinacho et al. (2014) **This study** Pinacho-Pinacho et al. (2014) Pinacho-Pinacho et al. (2014) Pinacho-Pinacho et al. (2014) Caspeta-Mandujano and Moravec (2000) Pinacho-Pinacho et al. (2014) Salgado-Maldonado et al. (2014) Caspeta-Mandujano and Moravec (2000) Pinacho-Pinacho et al. (2014) Salgado-Maldonado et al. (2014) **This study**
*Profundulus candalarius* Hubbs, 1924	**Digenea** *Clinostomum* sp. (M) *Phyllodistomum inecoli* (A) *Paracreptotrema blancoi* (A) **Cestoda** *Bothriocephalus acheilognathi* (A) **Nematoda** *Eustrongylides* sp. (L) *Spinitectus humbertoi* (A)	**This study** **This study** **This study** Velazquez-Velazquez et al. (2015) **This study** **This study** **This study**
*Profundulus guatemalensis* (Günther, 1866)	**Digenea** *Paracreptotrema blancoi* (A) **Monogenea** *Urocleidoides* sp. (A) **Cestoda** *Bothriocephalus acheilognathi* (A) **Nematoda** *Rhabdochona salgadoi* (A) *Spinitectus humbertoi* (A)	Salgado-Maldonado et al. (2014) **This study** **This study** **This study** Salgado-Maldonado et al. (2014) **This study** Salgado-Maldonado et al. (2014) **This study**
*Profundulus hildebrandi* Miller, 1950	**Cestoda** *Bothriocephalus acheilognathi* (A)	Velázquez-Velázquez et al. (2011) **This study**
*Profundulus kreiseri* Matamoros, Schaefer, Hernández & Chakrabarty, 2012	**Digenea** *Paracreptotrema blancoi* (A) **Nematoda** *Rhabdochona salgadoi* (A) *Spinitectus humbertoi* (A)	Salgado-Maldonado et al. (2014) **This study** Salgado-Maldonado et al. (2014) **This study** Salgado-Maldonado et al. (2014)
*Profundulus labialis* (Günther, 1866)	**Digenea** *Paracreptotrema blancoi* (A) **Nematoda** *Rhabdochona salgadoi* (A) *Spinitectus humbertoi* (A) *Spinitectus mariaisabelae* (A)	**This study** Salgado-Maldonado et al. (2011a) Salgado-Maldonado et al. (2014) Salgado-Maldonado et al. (2014) Salgado-Maldonado et al. (2011a)
*Profundulus oaxacae* (Meek, 1902)	**Digenea** *Paracreptotrema blancoi* (A) *Paracreptotrema profundulusi* (A) **Nematoda** *Rhabdochona salgadoi* (A)	Salgado-Maldonado et al. (2014) Salgado-Maldonado et al. (2011b) Salgado-Maldonado et al. (2014) Salgado-Maldonado et al. (2014)
*Profundulus portillorum* Matamoros & Schaefer, 2010	**Digenea** *Paracreptotrema blancoi* (A) Allocreadiidae gen. sp. (A) **Cestoda** *Bothriocephalus acheilognathi* (A) **Nematoda** *Rhabdochona salgadoi* (A) *Spinitectus humbertoi* (A) *Spiroxys* sp. (L)	**This study** **This study** Salgado-Maldonado et al. (2015) Salgado-Maldonado et al. (2014) **This study** **This study**
*Profundulus punctatus* (Günther, 1866)	**Digenea** Ascocotyle (Ascocotyle) felippei (M) *Centrocestus formosanus* (M) *Clinostomum* sp. (M) *Phyllodistomum inecoli* (A) *Paracreptotrema blancoi* (A) *Paracreptotrema profundulusi* (A) **Monogenea** *Urocleidoides* sp. (A) **Nematoda** *Contracaecum* sp. (L) *Eustrongylides* sp. (L) *Rhabdochona salgadoi* (A) *Spinitectus humbertoi* (A) *Spinitectus mariaisabelae* (A)	**This study** **This study** Salgado-Maldonado et al. (2011a) **This study** Pérez-Ponce de León et al. (2015) Salgado-Maldonado et al. (2011b) Salgado-Maldonado et al. (2014) **This study** Salgado-Maldonado et al. (2011b) Salgado-Maldonado et al. (2014) **This study** Salgado-Maldonado et al. (2011a) **This study** Salgado-Maldonado et al. (2011a) Salgado-Maldonado et al. (2014) Salgado-Maldonado et al. (2011a) **This study** Salgado-Maldonado et al. (2014) Caspeta-Mandujano et al. (2007) Salgado-Maldonado et al. (2011a)
*Profundulus* sp. 1	**Nematoda** *Rhabdochona salgadoi* (A) *Spinitectus humbertoi* (A)	Salgado-Maldonado et al. (2014) Salgado-Maldonado et al. (2014)
*Profundulus* sp. 2	**Digenea** *Phyllodistomum inecoli* (A) *Paracreptotrema blancoi* (A) *Paracreptotrema profundulusi* (A) **Nematoda** *Rhabdochona salgadoi* (A)	Pérez-Ponce de León et al. (2015) Salgado-Maldonado et al. (2014) Salgado-Maldonado et al. (2011b) **This study** Salgado-Maldonado et al. (2014) Salgado-Maldonado et al. (2011b) **This study** Salgado-Maldonado et al. (2014) Salgado-Maldonado et al. (2011a)

**Figure 3.**
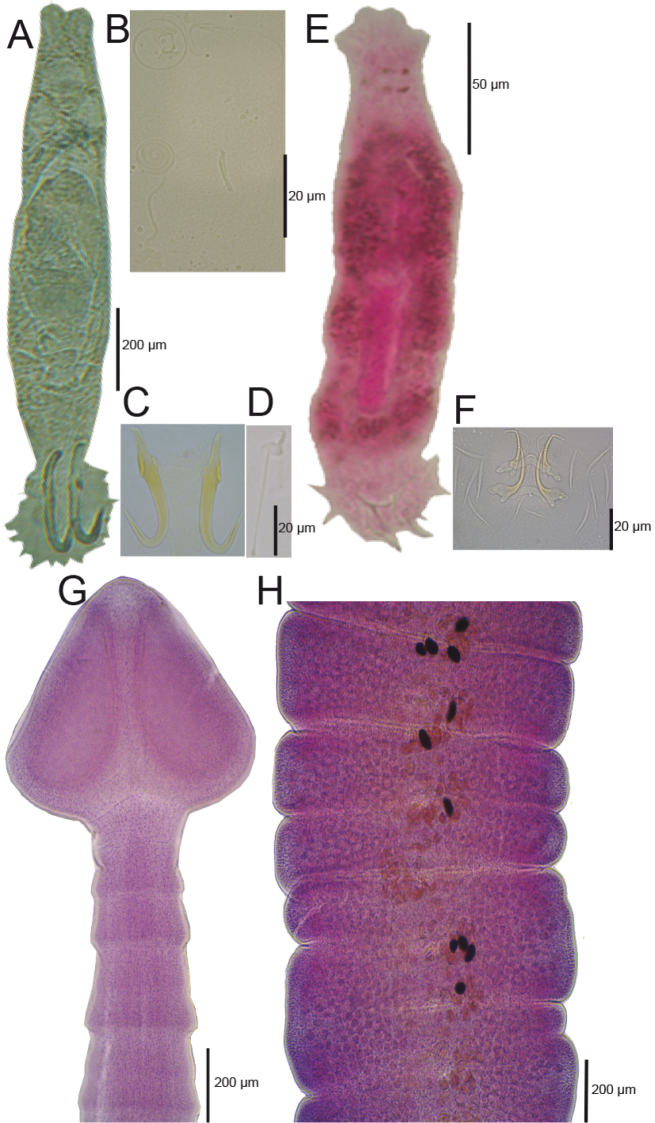
Species of monogeneans and the single cestode found in *Profundulus* spp. **A–D** *Gyrodactylus* sp. **E–F** *Urocleidoides* sp. **G**–**H** *Bothriocephalus acheilognathi*.

**Figure 4.**
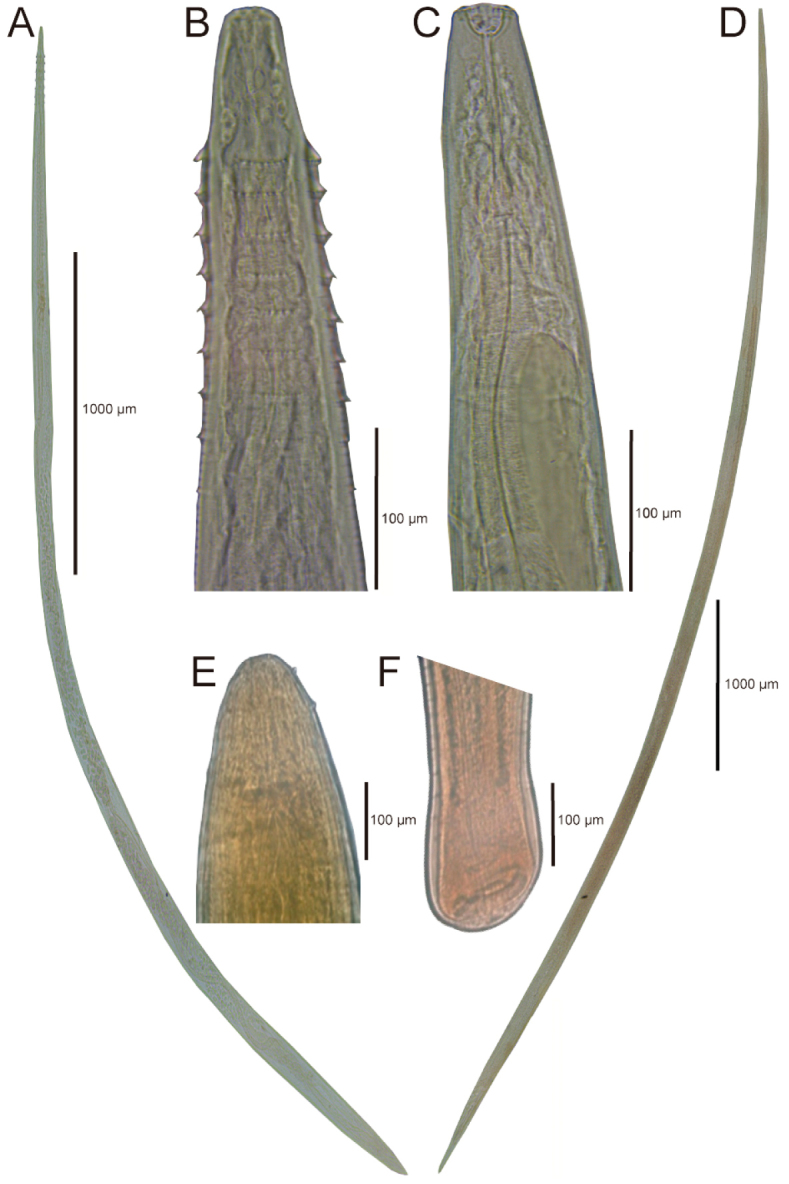
Species of nematodes found in *Profundulus*. **A**–**B** *Spinitectus humbertoi* **C**–**D** *Rhabdochona salgadoi* **E**–**F** *Eustrongylides* sp.

Digeneans exhibit the highest species richness (11 species), followed by nematodes (six species) and monogeneans (two species) (Table 2). Based on the observed host-specificity, at least six of the 12 adult helminth taxa listed in this work, i.e. *Paracreptotrema blancoi* *sensu* Salgado-Maldonado et al. (2011b), *Paracreptotrema profundulusi* Salgado-Maldonado, Caspeta-Mandujano & Martínez Ramírez, 2011, *Phyllodistomum spinopapillatum* Pérez-Ponce de León, Pinacho-Pinacho, Mendoza-Garfias & García-Varela, 2015, *Spinitectus humbertoi* Mandujano-Caspeta & Moravec, 2000, *Spinitectus mariaisabelae* Caspeta-Mandujano Cabañas-Carranza & Salgado-Maldonado, 2007 and *Rhabdochona salgadoi* Mandujano-Caspeta & Moravec, 2000, have only been recorded as parasites of profundulids and can be considered as members of the ‘core’ helminth fauna (in an historical biogeographical sense, not to be confused with the ecological “core-satellite” species concept, see Pérez-Ponce de León and Choudhury 2002). The cestode *Bothriocephalus acheilognathi* Yamaguti, 1934 has successfully infected some species of *Profundulus*; this is an introduced species that is commonly found in several freshwater fishes in North and Central America as a result of the introduction of cyprinids (carps) for aquaculture (see Choudhury et al. 2013). The digenean *Centrocestus formosanus* Nishigori, 1924 also represents a species that was introduced in North America, and perhaps Middle-America, through the introduction of its snail host, *Melanoides tuberculata* (Müller, 1774), from Asia (Scholz and Salgado-Maldonado 2000). Of the 20 taxa found, eight (40%) were larval forms of generalist species that use freshwater fish as intermediate or paratenic hosts. In seven of the eight species, fish-eating birds are the definitive hosts, and only one, *Spiroxys* sp., requires a different vertebrate to complete its life cycle. Adult nematodes of the genus *Spiroxys* Schneider, 1866 commonly occur in the digestive tract of freshwater turtles, but can also parasitize frogs, salamanders and snakes (Li et al. 2014). Larval forms have been reported from a wide spectrum of hosts in various localities in Middle-America (e.g. Aguirre-Macedo et al. 2001; Sandlund et al. 2010).

The most widely distributed parasites among profundulids are the nematodes *Rhabdochona salgadoi* and *Spinitectus humbertoi*, and the digenean *Paracreptotrema blancoi* *sensu* Salgado-Maldonado et al. (2011b), which are found in ten, eight and nine species of profundulids, in 38, 38 and 20 localities across Middle-America, respectively. Among these localities, prevalence and mean intensity values are quite variable (see Table 2). Prevalence varies between 2 and 100% for the different helminth species, although mean intensity values are usually very low (between one and five helminths per infected host), except for two larval forms, the heterophyids *Centrocestus formosanus* and Ascocotyle (Ascocotyle) felippei Travassos, 1928. These two species reached mean intensity levels as high as 821.6 and 165.4 larvae per infected host, respectively. Among adults, the nematodes *Spinitectus humbertoi* and *Rhabdochona salgadoi* reached mean intensity values usually higher than five worms per infected host among the various localities.

In terms of the species richness of the helminths in relation to the host species, *Profundulus balsanus* is the species with the highest diversity, since it is parasitized by 14 species, followed by *Profundulus punctatus* with 12, and *Profundulus candalarius* and *Profundulus portillorum* with six (Table 3). Finally, *Profundulus oaxacae*, *Profundulus kreiseri* and *Profundulus hildebrandi* possess a depauperate fauna, with only three, three and one species, respectively.

## Discussion

Fish were collected at 26 localities in southern Mexico, Guatemala and Honduras, and a total of 267 individual fish belonging to nine species of *Profundulus* was examined for helminth parasites. The inventory was completed by adding these records to the previous parasite surveys conducted on members of this fish group endemic to Middle-America. Interestingly, the number of individual hosts studied for helminths of this fish group has increased significantly during a two-year period across the entire distributional range, and it seems that only two new species were found. Firstly, a detailed morphological evaluation of the specimens recorded herein as *Urocleidoides* sp. indicate they represent an undescribed species, which will be formally described in a separate paper. A thorough revision of the morphology of the specimens identified as the trematode *Paracreptotrema blancoi* by Salgado-Maldonado et al. (2011b), along with the new samples obtained in this study, allowed us to determine that they in fact represent not only a new species but a new genus. The new species is readily distinguished by the size of the ventral sucker and by having a more restricted vitellarium, a shorter cirrus sac and caeca that bifurcate at the level of the ventral sucker and end at the level of the testes. Since information was also gathered from sequences of the 28S rRNA gene and scanning electron microscopy micrographs, the new species will be formally described in a separate paper. The record in this checklist is presented provisionally, under the original designation of the species, as *Paracreptotrema blancoi* *sensu* Salgado-Maldonado et al. (2011b).

Six adult helminth species are considered to be part of the biogeographical ‘core’ helminth fauna of profundulids. As discussed by Pérez-Ponce de León and Choudhury (2002), for a parasite taxon to be considered part of a biogeographical core, it must not only be widely distributed but must also be characteristically associated with and restricted to a monophyletic group of host species (see also Choudhury and Dick 1998), even if it is not present in all host species of that group. This concept was actually used to describe the pattern of host-specificity among the helminth parasite fauna of freshwater fishes in Mexico (Pérez-Ponce de León and Choudhury 2005); this was based on the premise that particular host-groups are characteristically associated with a biogeographical ‘core’ helminth fauna and that such host specificity strongly influences their biogeography. These authors tested three predictions based on that fundamental hypothesis of ‘core’ parasite faunas: (1) that the parasite fauna is largely circumscribed by higher levels of monophyletic host taxa (families, orders, etc.), and that this pattern is independent of areas; (2) that areas within a certain biogeographical region, and consequently with a similar fish composition, will have more similar parasite faunas compared with areas with a less similar fish faunal composition; and (3) that ‘core’ parasite faunas persist to some extent in transitional areas (areas where Nearctic and Neotropical species are sympatric) with limited host-sharing. The current results on the helminth fauna of *Profundulus* spp. along its distributional range in Middle-America further corroborate the three predictions.

This represents the second complete inventory of a freshwater fish group. Martínez-Aquino et al. (2014) recently published the inventory of the helminth parasites of goodeines, an endemic subfamily from central and a few areas of northern Mexico. Both groups belong in the order Cyprinodontiformes, and molecular phylogenetic analyses show that they are sister taxa (Webb et al. 2004, Doadrio and Domínguez 2004). Based on the premise that comprehensive data on the inventory of a particular host group is fundamental to a better understanding of the historical biogeography and evolutionary history of host-parasite associations, the information presented in this paper, along with the one for the goodeines, will allow us to discuss factors that have shaped the biogeographical and diversification patterns of parasites and hosts within a phylogenetic framework, and, to the best of our knowledge, this is the first time that these types of data have been produced.

There are, however, some notable differences between the biogeography of the Goodeinae and Profundulidae. The Goodeinae is an endemic fish component of central and northern Mexico which experienced an important adaptive radiation and contains 45 species (Domínguez-Domínguez et al. 2010). The helminth fauna of extant species (some of them have gone extinct recently due to habitat degradation) includes 51 species, according to the examination of almost 8,300 individual fish representing 36 species allocated to 18 genera, studied in 113 localities across central and northwestern Mexico (Martínez-Aquino et al. 2014). In contrast, *Profundulus* possesses only 11 species and is the only genus within the Profundulidae. These fish did not experience the same level of diversification as goodeines, and their distributional range comprises an area of Middle-America from central Mexico southwards to Honduras. Apparently, the Balsas depression establishes the distributional limit for both fish groups, since goodeines have the southernmost distribution range in the Balsas drainage, whereas profundulids reach their northernmost distribution in the same basin. However, these fish families do not occur sympatrically at any location. Since the Balsas River basin is the result of a geological event known as the Balsas Portal, which represents a marine transgression that occurred during the Mid-Cretaceous period (see Domínguez-Domínguez and Pérez-Ponce de León 2009, and references therein), it cannot be ruled out that this was the geological event that caused the divergence between goodeines and profundulids from a common ancestor, despite a molecular clock analysis showing that the ancestral split occurred during the Mid-Miocene, approximately 16 million years ago (see Doadrio and Domínguez 2004).

Irrespective of the biogeographical history of the ancestor of both profundulids and goodeids, and the subsequent radiation of the latter, the former did not diversify in the same way as goodeids did. Adaptive radiation of goodeines in central Mexico, following a complex geological and hydrological history (see Domínguez-Domínguez et al. 2010), resulted in a higher species richness, and this may have influenced their parasite fauna, contrasting the 51 helminth species that parasitize goodeines with only 20 species in profundulids. Interestingly, the helminth species composition in both host groups is relatively similar. The core helminth parasite fauna includes members of the Allocreadiidae Looss, 1902, Gorgoderidae Looss, 1901 and Haploporidae Nicoll, 1914 among the digeneans, members of the monogenean genus *Gyrodactylus* von Nordmann, 1832 and members of the nematode genus *Rhabdochona* Railliet, 1916. For instance, while goodeines are infected by two species of the allocreadiid genus *Margotrema* Lamothe-Argumedo, 1970, profundulids are infected by two species of the allocreadiid genus *Paracreptotrema* Choudhury, Pérez-Ponce de León, Brooks & Daverdin, 2006. Both host groups are parasitized by two species of *Phyllodistomum* Braun, 1899, and by two species of *Rhabdochona*. Likewise, scarce phylogenetic information is available to make strong comparisons, and a robust pattern cannot be established in the absence of a phylogenetic framework. However, the few available data show that the presence of congeners of different helminth groups in goodeines and profundulids is not the result of a historical association but of colonization (Brooks and McLennan 1993). In the morphological phylogenetic analysis of species of *Rhabdochona* by Mejía-Madrid et al. (2007), *Rhabdochona lichtenfelsi* Sánchez-Alvarez, García-Prieto & Pérez-Ponce de León, 1998 (a common and widely distributed parasite of goodeines) and *Rhabdochona salgadoi* (a common and widely distributed species in *Profundulus*) are not close relatives, although, needless to say, the phylogenetic analysis was not fully resolved and the morphology-based phylogeny may not be robust. In contrast, in the case of the allocreadiids, recently published molecular phylogenetic analyses clearly indicate that *Margotrema* spp. (in goodeines) and *Paracreptotrema* (in profundulids) are not sister taxa, since *Margotrema* clusters with species usually found in Nearctic fishes, whereas *Paracreptotrema* is the sister taxon to other allocreadiids that parasitize characids (Razo-Mendivil et al. 2014, a group of fish with a Neotropical origin, precluding any speculation about the speciation event that may have caused their diversification either in goodeines or profundulids).

As suggested by Pérez-Ponce de León and Choudhury (2010), molecular data are fundamental to better understanding patterns of diversity among the freshwater fish parasite fauna, but also to establishing sister group relationships among newly discovered species with respect to those already described. At present, it seems plausible to propose that the helminth fauna of goodeines was secondarily acquired from Nearctic fishes, whereas profundulids obtained their helminths from other Neotropical freshwater fishes, i.e. their parasites are the result of host-switching events following colonization from other, most probably unrelated, hosts. But this needs to be determined by proper molecular co-phylogenetic analyses. The data generated thus far will enable us to conduct such analyses in the near future and to contribute to a better understanding of the evolution and biogeography of the freshwater fish helminth parasite fauna.
